# Response From Crisis Text Line to “Commentary on ‘Protecting User Privacy and Rights in Academic Data-Sharing Partnerships: Principles From a Pilot Program at Crisis Text Line’”

**DOI:** 10.2196/64015

**Published:** 2025-01-22

**Authors:** Dena Trujillo

**Affiliations:** 1 Crisis Text Line New York, NY United States

**Keywords:** crisis, research, data privacy, research ethics, digital rights

As current chief executive officer (CEO) of Crisis Text Line, I am writing this commentary on behalf of the organization to clarify the record, provide context, and supply background information regarding Crisis Text Line and its research standards regarding a critique [1] of a viewpoint paper [2] published in the *Journal of Medical Internet Research* in 2019. Below, you will find information about how we protect and do not commercialize personal data; our ethical research standards; our unique data; texter digital rights, privacy, and ethics; and a snapshot of our recent research.

## Crisis Text Line: Background

Crisis Text Line is a nonprofit organization that provides free, 24-7, confidential text-based mental health support and crisis intervention in both English and Spanish for anyone in the United States and Puerto Rico. Since our launch in 2013, we have supported nearly 10 million conversations in the United States and have trained over 71,000 volunteers.

All our volunteers are required to accept our policies as a condition of serving as a volunteer with Crisis Text Line, which disclose that we may share anonymized data with researchers. That disclosure and the consent to our all-encompassing privacy policy existed during and prior to the 2016-2017 period of the research that is the subject of the *Journal of Medical Internet Research* study at issue. Volunteers’ consent to that broader, generally applicable policy remains effective regardless of our decision to create a shorter and more tailored volunteer-specific policy in 2021 in the interest of greater transparency. Volunteers serve without compensation and are not under any employment agreements.

## Crisis Text Line Does Not Commercialize Personal Data

Crisis Text Line does not and has never monetized personally identifiable information directly or indirectly.

Crisis Text Line previously had an open and public relationship with Loris.ai, a for-profit company aimed at helping other for-profit companies employ de-escalation techniques to reduce stress for customers and employees. At the time, we believed that sharing insights from Crisis Text Line’s training curriculum and deidentified data could support stress reduction and improved well-being in spaces beyond the crisis context while potentially contributing to the sustainability of a nonprofit public good. The world of technology and data ethics evolved rapidly, and Crisis Text Line quickly changed course. Loris.ai did not access any Crisis Text Line anonymized data or other information after early 2020.

Public records demonstrate that Loris.ai *did not exist* during the 18-month period of the pilot analyzed in the study; the company was formed months after the pilot ended. Crisis Text Line severed all ties with Loris.ai in 2022 and required the return or deletion of any data previously accessed. We were publicly transparent about the goals of this relationship, which included broader application of our de-escalation strategies and potential revenue to support our important nonprofit work. Ultimately, Crisis Text Line did not receive financial gain as a result of its prior data- and insights-sharing relationship with Loris.ai.

Crisis Text Line is a nonprofit, and those acting as advisory board members did so on a voluntary unpaid basis. To Crisis Text Line’s knowledge, there was no conflict of interest, and none of the research authors were advisory board members for the commercial spin-off Loris.ai.

## The Critical Need for Ethical Research in Combating the Mental Health Crisis

We can all agree that the need for evidence-based action on the current mental health crisis is indisputable and urgent. The importance of ethical research focused on tackling this crisis cannot be overemphasized. The prevalence of mental health challenges worldwide highlights the critical need for innovative solutions, compassionate care, and evidence-based interventions. Through ethical research efforts, Crisis Text Line contributes to destigmatizing mental health challenges by disseminating accurate information, challenging misconceptions, and promoting empathy and acceptance. Through collaborative efforts [3] between researchers, policy makers, clinicians, and community stakeholders, we help to foster a holistic approach to mental health that addresses systemic barriers and promotes equitable access to care.

## In Pursuit of Solutions

Crisis Text Line’s anonymized data provide a unique snapshot of mental health across the United States. We are in direct conversation with people in crisis, so what we learn is not simply the result of a survey. Rather, it is real-time insight about how people feel. We ask about the specifics of their crisis, listen, collaboratively problem solve, de-escalate, and then provide them with an optional anonymous postconversation survey. Data shared by texters themselves in the optional postconversation survey suggest that two-thirds of them do not have access to professional mental health support. We believe it is our duty to elevate their voices and support research efforts that might contribute solutions to help with this emergency in an ethical manner. Analyzing that data—carefully secured and anonymized—allows us to observe emerging trends, almost in real time. For example, in March 2020 during the first COVID-19 lockdowns, we observed a spike in conversation volume as people reached out while struggling with isolation, anxiety, and grief, but with lower suicidal ideation. Our dataset of nearly 10 million conversations (nearly 300 million messages) collected in over 10 years of operation offers a broad and deep perspective of mental health trends across the country. Those data position us to have a real impact through ethical research endeavors.

## Data Privacy, Digital Rights, and Ethics

At Crisis Text Line, safeguarding our texters’ data is paramount. In moments of crisis, our texters can feel assured, trusting the integrity of the support they receive without concerns about their data’s privacy, security, or use. To that end, we’ve published digital rights [4] for texters, outlining rights and principles that we uphold throughout their use of our service. Ensuring texters’ safety, privacy, and confidentiality stand as foundational principles of our service. We protect texter privacy rigorously and maintain texter anonymity, except when necessary for safety or legal reasons.

Our privacy policy [5] transparently discloses how we collect and use data, noting our use of encryption and other reasonable measures to protect against unauthorized access. All texters get a link to the policy at the start of every conversation with us, and texters must consent to the policy to use the service. The policy also intentionally contains simple plain-language bullets at the beginning so a user can quickly and easily understand what we do, how we do it, and why—including, among other things, our use of anonymized deidentified data for research. Texters also have the option to ask that we delete their data, including conversation records and contact information, simply by texting or messaging the word DELETE.

We go to great lengths to protect, store, analyze, and share insights from our anonymized crisis conversations to ethically help the world address mental health issues. We further align with the highest international standards for ethical research involving human subjects. In addition to ensuring that external research collaborations are overseen by the institutional review board (IRB) of the lead researcher’s institution, since 2023, our in-house research is also overseen by an IRB. These policies and practices disclose and reinforce our commitments to upholding texter privacy and fulfilling our mission.

## Our Recent In-House and Collaborative Research

Since our launch in 2013, we have supported nearly 10 million conversations initiated by texters across the United States. Leveraging this anonymized and deidentified dataset, we inform policy makers, journalists, and researchers. Alongside our internal research endeavors, we foster collaborations with partners at research institutions. Recent examples of research contributing unique insights to the field include:

- Crisis Text Line & Common Good Labs: “What Young People in Crisis Need From Their Communities” (2023) [6]
- Crisis Text Line’s United in Empathy Report (2023) [7]
- Examining Hurricane Ida’s Impact on Mental Health: Results From a Quasi-Experimental Analysis (2023) [8]
- The Use of Crisis Services Following the Mass School Shooting in Uvalde, Texas: Quasi-Experimental Event Study (2023) [9]
- Crisis Response Among Essential Workers and Their Children During the COVID-19 Pandemic (2021) [10]

## A Call to Action

As we navigate the complexities of the mental health crisis, we reaffirm our commitment to ethical research as a catalyst for change. We embrace the transformative power of collaborative, ethical, impactful research in illuminating the path toward solutions, healing, and hope.

## Abbreviations

CEO: chief executive officer
IRB: institutional review board
